# Effect of Resin Content on the Mechanical and Ballistic Performance of B4C/Aramid III/UHMWPE Ceramic Composite Armor with Comparative Evaluation of Fiber Architecture and Manufacturing Process

**DOI:** 10.3390/ma19194144

**Published:** 2026-09-28

**Authors:** Yue Wu, Jie Zhang, Jianqiang Guo, Shaohua Wang, Fuping Li, Boming Zhang, Ronghai Wu

**Affiliations:** 1 School of Materials Science and Engineering, Beihang University, Beijing 100191, China; 2 Beijing Institute of Aeronautical Materials, Beijing 100095, China; guojianqiang2010@163.com (J.G.);; 3 State Key Laboratory of Solidification Processing, Northwestern Polytechnical University, Xi’an 710072, China

**Keywords:** aramid III/epoxy composites, resin content, mechanical properties, ceramic composite armor, ballistic performance, fracture cone

## Abstract

This study investigates the effects of resin content, fiber architecture, and manufacturing process on the mechanical response and ballistic behavior of Aramid III/epoxy support layers used in B4C ceramic composite armor. Three plain-woven Aramid III/epoxy laminates containing 20 wt%, 30 wt%, and 38 wt% resin were fabricated and characterized in terms of density, porosity, flexural properties, interlaminar shear strength, and low-velocity impact response. In addition, unidirectional (UD) and plain-woven laminates with a fixed resin content of 30 wt% were compared to clarify the influence of fiber architecture, while autoclave and hot-press processing were compared in terms of internal porosity and structural uniformity. The results showed that the 30 wt% resin laminate exhibited the lowest measured porosity (0.3%) and the best overall balance of flexural strength, flexural modulus, and impact-energy absorption, with values of 421 MPa, 34.6 GPa, and 141.5 J, respectively. The UD laminate exhibited a 38.5% higher impact-energy absorption than the corresponding plain-woven laminate, demonstrating the influence of continuous fiber alignment on load transfer and impact deformation. The autoclave process produced lower porosity than hot pressing for the 30 wt% laminate, indicating improved consolidation and microstructural uniformity. Ballistic tests were conducted on the plain-woven support-layer configurations using 12.7 mm armor-piercing incendiary projectiles under identical nominal areal density and impact conditions. The 30 wt% configuration achieved protection in all four tests and exhibited a mean back-face signature of 11.25 mm, compared with protection success rates of 50% and 75% for the 20 wt% and 38 wt% configurations, respectively. Post-impact characterization revealed B4C fragmentation, fiber deformation, matrix cracking, interlaminar delamination, and deformation of the UHMWPE backing layer. These results indicate that an intermediate resin content provides a favorable balance between interfacial load transfer and fiber deformation within the investigated armor configuration. The effects of fiber architecture and manufacturing process are also discussed as important factors affecting the structural and mechanical response of the support layer.

## 1. Introduction

With the increasing complexity of modern battlefield environments and the growing demand for lightweight aerospace systems, lightweight composite armor featuring both high specific strength and strong protection capability has become a major research focus in the field of personal and vehicle protection. Although conventional metallic armor offers high strength and good structural stability, its relatively high areal density limits its use in applications requiring high mobility and weight reduction, such as attack helicopters, armored vehicles, and naval platforms [1]. In contrast, ceramic/fiber composite armor combines the high hardness of ceramics with the high toughness of fiber-reinforced composites, making it one of the most promising approaches for lightweight ballistic protection.

Typical ceramic composite armor consists of a hard ceramic strike face and a fiber-reinforced composite support layer. The ceramic layer primarily dissipates the initial impact energy by eroding the projectile core, inducing projectile deflection, and forming a fracture cone, whereas the backing layer confines ceramic fragments, suppresses crack propagation, and absorbs residual energy to provide synergistic protection [2,3,4]. Previous studies have shown that the stiffness, interfacial bonding state, and deformation capacity of the support layer have a significant influence on the fracture pattern of the ceramic layer and on the overall ballistic resistance of the armor system [4,5]. Yu et al. [6] reported that increasing the stiffness of the backing plate can enlarge the ceramic fracture-cone region, thereby enhancing energy dissipation in the ceramic layer. Wu et al. [7] further demonstrated that interfacial damage alters stress-wave propagation and penetration failure modes in ceramic composite armor systems.

For fiber-reinforced laminates, the effectiveness of the support layer is governed not only by its initial flexural stiffness and strength, but also by the stability of load transfer between adjacent plies. Interlaminar damage, including matrix cracking, delamination, and local debonding, can progressively reduce the effective stiffness of a laminate and modify its deformation and load-transfer characteristics. In particular, stiffness degradation under repeated or severe loading can alter the ability of a composite support layer to constrain a ceramic strike face and redistribute transient impact loads. Blythe et al. [8] demonstrated that the stiffness degradation of hybrid fiber laminates under cyclic three-point bending is closely associated with the development of damage within the laminate, highlighting the importance of considering both the initial mechanical properties and damage evolution when evaluating composite structural performance. Therefore, optimization of a ceramic armor backing layer should consider the coupled effects of resin content, interfacial integrity, fiber architecture, and damage evolution rather than relying solely on a single initial mechanical-property parameter.

In recent years, extensive research has been devoted to high-performance fiber composites. Aramid fibers and ultra-high-molecular-weight polyethylene (UHMWPE) fibers are widely used in lightweight protective structures because of their excellent impact resistance [6,7,9]. Qu et al. [10] studied the influence of elastic modulus and thickness ratio on the damage mechanisms of aramid/UHMWPE composite target plates; while Wang et al. [11] showed that aramid fabrics modified with polyethylene and graphene exhibit significantly improved ballistic performance. Kazemi et al. [12] proposed a zoned hybrid laminate architecture that effectively enhances the energy-absorption capability of composites under high-velocity impact conditions. Meanwhile, increasing attention has been paid to the influence of fiber architecture on impact resistance. Previous studies have shown that unidirectional (UD) structures can effectively reduce stress concentrations associated with fiber interlacing points, thereby improving the efficiency of fiber participation in cooperative deformation and enhancing impact-energy absorption performance [11,12,13,14].

Regarding ceramic composite armor, numerous experimental and numerical investigations have recently been conducted in China concerning the penetration behavior of 12.7 mm armor-piercing incendiary projectiles. Zhu et al. [15] numerically investigated the penetration behavior of 12.7 mm API projectiles against ceramic composite armor systems composed of different constituent materials. Yang et al. [16] studied the penetration performance of ZrO_2_-toughened Al_2_O_3_ ceramic composite projectiles against ceramic armor. Tan et al. [17] analyzed the ballistic-limit velocity and multi-hit resistance of ceramic composite armor systems. Feng et al. [18] examined the influence of projectile impact angle on the penetration behavior of ceramic composite armor. Lu et al. [19] further investigated the effects of ceramic-thickness ratio and fiber-reinforced polymer backing plates on ballistic performance and damage evolution. However, despite these efforts, systematic studies investigating how resin content regulates the interfacial state and mechanical properties of support layers, thereby influencing ceramic fragmentation behavior and overall ballistic performance, remain relatively limited. In particular, quantitative relationships among backing-layer properties, ceramic fracture behavior, and global protective efficiency have not yet been comprehensively established.

For protection against 12.7 mm armor-piercing incendiary (API) projectiles, the mainstream armor design typically adopts a three-layer architecture. In such systems, high-hardness and high-flexural-strength ceramics, such as B4C, are employed as the strike face to fracture and erode the projectile. Behind the ceramic layer, high-modulus fiber-reinforced polymer composites are used as support layers to provide structural confinement and enable the ceramic to fully exploit its superior compressive properties [20,21,22]. Finally, fiber-reinforced polymer composites with high tensile strength and elongation, such as UHMWPE composites, are incorporated as energy-absorbing backing layers to dissipate the residual kinetic energy of the projectile and ultimately prevent perforation. During ballistic impact, the ceramic layer typically accounts for more than 60% of the total energy absorption, and its protective efficiency is closely related to the stiffness of the support layer. Previous studies have shown that when low-stiffness materials are directly combined with ceramics, the degree of ceramic fragmentation is significantly reduced, resulting in less effective projectile erosion and fracture [23,24,25]. Early ceramic composite armor systems commonly employed carbon-fiber-reinforced laminates as support layers; however, carbon-fiber composites exhibit limited energy-absorption capability. In contrast, polyurethane-based aramid and UHMWPE composites, which are widely used in ballistic protection, possess excellent energy-absorption characteristics but generally exhibit flexural strengths of only 100–200 MPa. Consequently, they are unable to provide sufficient support for the ceramic strike face, preventing the ceramic layer from achieving its full protective potential. To address this limitation, increasing attention has been devoted in recent years to aramid/epoxy composites as support-layer materials. By introducing an epoxy matrix, these composites can simultaneously achieve enhanced flexural strength, increased stiffness, and improved energy-absorption capability, thereby enabling lightweight and high-performance armor designs. Among the various design parameters, resin content plays a critical role in determining the support performance of the composite. Insufficient resin content leads to weak interfacial bonding between fibers and inadequate structural stiffness, whereas excessive resin content restricts fiber deformation during ballistic impact and increases the tendency for shear-dominated failure. Therefore, a rational optimization of resin content, based on the interaction mechanisms between the projectile and the armor system during penetration, is essential for maximizing the performance of the ceramic strike face and improving the overall ballistic resistance of ceramic composite armor systems [26,27,28].

Despite these advances, a systematic understanding of the coupled effects of support-layer composition, fiber architecture, and manufacturing process on the mechanical and ballistic response of ceramic composite armor remains limited. In particular, resin content can simultaneously influence fiber impregnation, interfacial load transfer, laminate stiffness, and fiber deformation, while fiber architecture and manufacturing conditions may further modify stress redistribution and damage evolution.

Accordingly, the objectives of this study are to: (i) investigate the influence of resin content on the microstructure, flexural properties, interlaminar shear strength, and impact-energy absorption of Aramid III/epoxy laminates; (ii) compare UD and plain-woven architectures to clarify the effect of fiber architecture on the mechanical and impact response; (iii) evaluate the influence of autoclave and hot-press processing on laminate porosity and structural uniformity; and (iv) determine how the resulting support-layer characteristics affect ceramic fracture, support-layer damage, back-face deformation, and ballistic protection within a B4C/Aramid III/UHMWPE armor system subjected to 12.7 mm API impact. The ballistic investigation focuses on plain-woven support layers with different resin contents, whereas the effects of fiber architecture and manufacturing process are evaluated primarily through material-level characterization.

Because only three resin contents and one armor architecture were investigated, the optimum resin content identified in this study is considered specific to the investigated material system and loading conditions rather than a universal optimum for ceramic composite armor.

## 2. Experimental Section

### 2.1. Raw Materials

The reinforcement used was plain-woven Aramid III fabric with an areal density of 200 g/m^2^ and a fiber diameter of approximately 12 μm. Unidirectional (UD) Aramid III composites were also prepared for comparative analysis. The matrix was a toughened bisphenol-A epoxy resin system featuring a glass transition temperature (*T_g_*) of approximately 180 °C. The ceramic layer consisted of hot-pressed sintered B_4_C tiles with a thickness of 10 mm, a density of 2.55 g/cm^3^, and a Vickers hardness of 34 GPa. UHMWPE laminates were employed as the final energy-absorbing layer, and ethylene-vinyl acetate (EVA) adhesive films were used for interlaminar bonding.

### 2.2. Characterization of the Resin System

Non-isothermal curing analysis of the resin system was performed using a differential scanning calorimeter (The thermal properties were characterized using a DSC 214 Polyma differential scanning calorimeter (NETZSCH-Gerätebau GmbH, Selb, Germany)) at a heating rate of 10 °C/min. Figure 1a shows the DSC thermogram of the toughened epoxy resin. The curing exothermic peak of the 615-3 resin initiates at approximately 160 °C, establishing the nominal curing temperature at 180 °C.

The viscosity–temperature relationship of the resin system was evaluated using a rotational rheometer (MCR 302e, Anton Paar, Graz, Austria). as shown in Figure 1b. With increasing temperature, the resin viscosity dropped rapidly, falling below 0.1 Pa·s at around 130 °C, which benefits the thorough infiltration of the resin into the internal Aramid III fiber bundles. Beyond 160–170 °C, cross-linking initiation triggers a sharp increase in viscosity [29].

### 2.3. Fabrication Process of Aramid III Composites

Aramid III composites were manufactured via a prepreg-autoclave process. The autoclave provides a highly uniform pressure field that minimizes porosity, serving as the benchmark standard for high-performance composite manufacturing. By adjusting the resin film configuration, three batches with resin contents of 20 wt%, 30 wt%, and 38 wt% were obtained, denoted as Groups A, B, and C, respectively. The curing cycle proceeded as follows: heating to 130 °C at 3 °C/min and holding for 30 min to ensure full fiber wetting, heating to 150 °C and holding for 60 min, and finally, heating to 180 °C and holding for 90 min to achieve complete cross-linking [30].

### 2.4. Characterization of Microstructure and Mechanical Properties

(1) The bulk density and porosity of the composites were measured using the Archimedes method. Ultrasonic C-scan and micro-CT were conducted to inspect internal defects and trace impact damage evolution [31].
(2) Three-point bending tests were carried out in accordance with ASTM D7264. The specimen dimensions were 80 mm ×15 mm × 4 mm with a support span of 64 mm and a loading crosshead speed of 2 mm/min. The flexural strength (*σ_f_*) and flexural modulus (*E_f_*) were calculated via Equations (1) and (2) [32]:
  (1) $$\sigma_{f}=\frac{3P_{\max}L}{2bh^{2}}$$
  (2) $$E_{f}=\frac{L^{3}}{4bh^{3}}\cdot \frac{\Delta P}{\Delta d}$$
  where *P_max_* is the maximum load, *L* is the span length, *b* and *h* are the width and thickness of the specimen, and $\frac{\Delta P}{\Delta d}$ represents the slope of the initial linear portion of the load–displacement curve.
(3) Short-beam shear tests were conducted as per ASTM D2344 to determine the interlaminar shear strength (ILSS) using Equation (3) [33,34]:
  (3) $$\mathrm{ILSS}=\frac{3P_{\max}}{4\mathrm{bh}}$$
(4) Drop-weight impact testing was conducted using an Instron Dynatup 8250HV impact tester (Instron, Norwood, MA, USA) following ASTM D7136. The target plates measured 100 mm × 100 mm × 4 mm, supported by a clamping ring with an inner diameter of 76 mm. The impactor mass was 5.4 kg with a hemispherical striker diameter of 12.7 mm. The peak load, maximum displacement, and absorbed energy (*E_abs_*) were determined from the load–displacement profiles, where the total absorbed energy was integrated using Equation (4) [35,36,37]:
  (4) $$E_{\mathrm{abs}}=\int_{0}^{d_{\max}}F(d)\mathrm{dd}$$
  where *d_max_* is the maximum impactor displacement.

### 2.5. Armor Assembly and Ballistic Testing

The structural configuration and ballistic protection sequence of the armor are illustrated schematically in Figure 2. A “sandwich” architecture was configured, consisting sequentially of a 10 mm thick B_4_C ceramic faceplate, an Aramid III composite support backing (Options A, B, or C), and a 5 mm thick UHMWPE energy-absorbing backing. EVA adhesive films were placed at each interface. Square targets measuring 100 mm × 100 mm were co-cured in an autoclave run to ensure high interlaminar bonding quality. The total nominal areal density of the target systems was tightly restricted to 42.5 ± 0.1 kg/m^2^.

Ballistic trials were conducted at a professional range according to the GJB 59.18-1988 standard. Type-54 12.7 mm API projectiles (hardened steel core, mass 30.8 g) were launched via a ballistic gun. Post-impact, the protective outcome (complete protection vs. perforation) and the back face signature (BFS, representing the rear deformation depth) were recorded for each target [25,38,39].

The present study was designed as a controlled investigation of resin content within a fixed ceramic composite armor architecture. The resin contents were selected as 20, 30, and 38 wt%, while the B4C ceramic thickness, UHMWPE backing thickness, nominal areal density, target dimensions, and projectile type were kept constant. This controlled design was adopted to isolate the influence of the Aramid III/epoxy support-layer formulation on ballistic response. The experimental matrix does not encompass multiple ceramic thicknesses, projectile calibers, areal densities, or armor architectures. Therefore, the optimum resin content identified in this work should be interpreted within the investigated configuration and loading conditions.

### 2.6. Failure Morphology Analysis

Post-impact targets were carefully sectioned and disassembled to perform failure mode characterization:

(1) Ceramic Faceplate: High-resolution digital imaging was performed on the rear faces of the shattered ceramic plates. The schematic partition of the ceramic fragmentation cone is detailed in Figure 3. The top diameter (*D*) and the bottom major crack ring diameter (*d*) of the fragmentation cone were measured, and the semi-cone angle (*α*) was computed via Equation (5):
  (5) $$\alpha =\arctan\left(\frac{D-d}{2h}\right)$$
  where *h* is the ceramic thickness. Additionally, the number of primary radial cracks propagating from the impact center was statistically counted.
(2) Composite Support Backing: Macro-photography was conducted on the strike and rear faces of the support layers to chart the penetration depth, damage zone boundary, and fiber delamination area.
(3) UHMWPE backing: The deformation profiles and localized failure characteristics were systematically examined.

### 2.7. Statistical Analysis

Statistical analysis was performed to quantify the variability and statistical significance of the experimental results. Continuous variables are reported as mean ± standard deviation (SD). For the ballistic tests, the back-face signature (BFS) was calculated only for targets that successfully arrested the projectile, whereas protection and perforation were treated as binary categorical outcomes. Because of the limited number of ballistic tests in each configuration, two-sided Welch’s *t*-tests were used for pairwise comparisons of BFS, and Fisher’s exact test was used for comparisons of protection rates. For multiple pairwise comparisons, the Holm correction was applied to control the family-wise error rate. For the ceramic fragmentation cone angle and radial crack count, one-way analysis of variance (ANOVA) was initially performed to evaluate the overall differences among the three resin-content groups, followed by pairwise comparisons with Holm correction. A probability value of *p* < 0.05 was considered statistically significant. Owing to the relatively small sample size, the statistical results are interpreted as exploratory evidence of group differences rather than definitive estimates of population-level performance.

## 3. Results and Discussion

### 3.1. Effect of Resin Content on Microstructure

Table 1 summarizes the experimental density and porosity of the composites with varying resin contents. Group B (30 wt% resin) displayed the lowest porosity (0.3%), indicating that within the three investigated formulations, this resin content provided the most favorable measured consolidation state. Group A (20 wt%) exhibited an elevated porosity of 1.8% due to insufficient resin volume, leaving micro-voids inside the dry fiber bundles. Conversely, Group C (38 wt%), despite being fully saturated, showed a porosity of 1.2%, suggesting that excessive resin content did not further reduce the measured void fraction under the present processing conditions [40].

Under identical fiber–resin ratios, specimens were fabricated via both autoclave and hot-press molding to assess processing efficiency. Figure 4 illustrates the ultrasonic C-scan maps of the samples. No major macroscopic defects were detected in either the autoclave or hot-press 30 wt% variants (in contrast, the 20 wt% variant showed severe resin starvation and local heterogeneity). However, the autoclave-molded samples manifested a deeper, more uniform color profile, which qualitatively suggests a more homogeneous internal structure.

This observation is corroborated by the micro-CT reconstructions in Figure 5. The rendered volumes show that the hot-press sample exhibited a higher measured void content (1.3%) than the autoclave-molded counterpart (0.3%), indicating more pronounced internal porosity under the investigated processing conditions.

Figure 6 compares the quasi-static mechanical properties of the composites molded by the two methods across different temperatures. At −40 °C, the puncture energy absorption of the autoclave laminate dropped slightly below that of the hot-press laminate. This occurs because the low-porosity autoclave laminate features rigid interlaminar bonding, and in a sub-zero environment, the highly embrittled epoxy matrix forces cracks to cut directly through both the fibers and matrix via trans-crystalline fracture. This shortened failure path limits macro-energy absorption. The hot-press material, featuring higher porosity, triggers more extensive interlaminar delamination and micro-crack branching, which paradoxically enhances its cryogenic damage tolerance and energy dissipation. Overall, however, the autoclave molding process outperforms hot-press molding in terms of ambient flexural strength and stiffness metrics.

### 3.2. Mechanical and Impact Properties

#### 3.2.1. Flexural and Interlaminar Shear Properties

The flexural strength, flexural modulus, storage modulus, ILSS, and drop-weight impact energy absorption of the composites are plotted in Figure 7. At a resin content of 30 wt% (Group B), the composite registered its peak flexural performance, with a flexural strength of 421 MPa and a flexural modulus of 34.6 GPa.

Group A (20 wt%) exhibited insufficient matrix impregnation, which may result in incomplete stress transfer between neighboring fibers and localized fiber sliding under bending. In contrast, although Group C (38 wt%) exhibited a relatively high ILSS of 28.1 MPa, the increased matrix fraction may constrain fiber deformation and promote matrix-dominated damage. Group B therefore provides the most favorable balance between fiber content and interfacial cohesion under the investigated conditions.

These observations are consistent with the established damage behavior of fiber-reinforced polymer laminates, in which matrix cracking, fiber–matrix debonding, and interlaminar delamination can progressively modify load-transfer paths and laminate stiffness [8]. The present results therefore suggest that the intermediate resin content provides a favorable balance between interfacial load transfer and fiber deformation, rather than directly establishing a universal optimum for all laminate systems.

#### 3.2.2. Drop-Weight Impact Performance and Failure Modes

Figure 8 illustrates the macro-failure morphology of the laminates following drop-weight impact. Group A exhibits severe fiber pull-out, extensive delamination, and localized penetration damage. Owing to the insufficient resin content, the interfacial bonding between fibers and matrix is relatively weak, limiting effective load transfer. Consequently, the observed damage morphology suggests that impact energy dissipation was associated with fiber rupture and localized delamination, resulting in poor resistance to impact loading. In contrast, Group B exhibited the highest impact resistance among all investigated configurations. As shown in Figure 8b, a prominent permanent indentation was formed without complete perforation, indicating that the impact load was effectively redistributed throughout the laminate. The moderate resin content provided sufficient interfacial bonding while maintaining the deformation capability of the fiber bundles, which is consistent with the observed combination of large deformation and high absorbed energy. Group C exhibited a typical brittle failure mode characterized by a distinct cross-shaped matrix cracking pattern. The excessive resin content increased the laminate stiffness but restricted fiber deformation, which was accompanied by more pronounced matrix-dominated cracking in the observed failure morphology.

The drop-weight results further confirm that Group B achieved the highest absorbed energy (141.5 J). The observed failure morphologies indicate that fiber deformation, matrix damage, interlaminar delamination, and frictional interactions may all have contributed to the measured energy absorption. However, the present experiments did not directly measure the individual energy contributions of these mechanisms. Therefore, these mechanisms are discussed as qualitative interpretations of the observed damage morphology rather than quantitatively resolved energy-partition results.

#### 3.2.3. Influence of Fiber Weaving Architecture

Figure 9 compares the mechanical properties of Aramid III unidirectional (UD) laminates and plain-woven fabric laminates at a fixed resin content of 30 wt%. The UD composite exhibited superior measured performance in all evaluated properties, with the most significant improvement observed in impact energy absorption, which increased by 38.5% to 196 J. The superior performance of the UD laminate can be attributed primarily to the continuous and straight alignment of the fibers. In contrast to woven fabrics, where fiber crimp and interlacing introduce local geometric distortions, the UD architecture minimizes stress concentrations and allows external loads to be transferred more directly along the fiber direction. Consequently, a larger proportion of fibers can be effectively engaged during loading, resulting in improved load-transfer efficiency and enhanced resistance to damage initiation. Under impact loading, the absence of crimp-related stress concentrations enables the UD laminate to mobilize a greater volume of longitudinal fibers to undergo tensile deformation and absorb impact energy. In addition, the straight fiber arrangement facilitates more uniform stress-wave propagation throughout the laminate, promoting the participation of surrounding fibers in energy dissipation. As a result, impact energy is distributed over a larger area rather than being concentrated near the impact site.

This behavior is further supported by the post-impact rear-face morphologies shown in Figure 10. The UD composite exhibited a broad and relatively uniform deformation zone, indicating extensive fiber participation and effective stress redistribution. In contrast, the woven laminate displayed a more localized failure region, suggesting that deformation is constrained by yarn interlacing and associated stress concentrations. Therefore, the UD architecture showed higher mechanical and impact performance at the coupon level under the present test conditions. Importantly, the ballistic armor tests reported in Section 3.3 employed the plain-woven Aramid III support layer; therefore, the present study does not provide a direct experimental comparison of the ballistic performance of UD and plain-woven architectures.

### 3.3. Global Ballistic Performance of Ceramic Composite Armor

#### 3.3.1. Ballistic Protection Efficiency

The live-fire ballistic test matrix and corresponding results for the three armor configurations are summarized in Table 2. Summary of live-fire ballistic test results for the ceramic composite armors. To further quantify the variability of the ballistic response, the protection rate and BFS statistics are summarized in Table 3, Table 4 and Table 5. To ensure a fair comparison, all armor systems were designed with closely matched areal densities and tested under identical impact conditions using 12.7 mm armor-piercing incendiary (API) projectiles. Each configuration was subjected to four replicate tests.

As shown in Table 2. Summary of live-fire ballistic test results for the ceramic composite armors, the armor system incorporating the Group B support layer exhibited the best ballistic performance, achieving a 100% protection success rate. In contrast, the Group A and Group C configurations achieved success rates of only 50% and 75%, respectively. However, Fisher’s exact tests showed no statistically significant differences in protection rate between any pair of groups (Table 5), indicating that the observed differences in binary protection outcome should be interpreted cautiously given the small sample size.

For non-perforated targets, the mean BFS values were 37.00 ± 5.66 mm for Group A, 11.25 ± 2.50 mm for Group B, and 24.33 ± 2.52 mm for Group C (Table 3). One-way ANOVA indicated a significant overall difference among the three groups (F = 43.739, *p* = 0.000264). Pairwise Welch’s *t*-tests with Holm correction showed a significant difference only between Groups B and C (adjusted *p* = 0.0048), whereas the differences between Groups A and B (adjusted *p* = 0.1495) and between Groups A and C (adjusted *p* = 0.1618) were not statistically significant (Table 4). These results suggest that the 30 wt% formulation was associated with the lowest rear deformation within the investigated configuration, but the available ballistic sample size does not support a general statistical claim that Group B has a universally superior ballistic performance.

The superior observed response of Group B is nevertheless consistent with the mechanical and microstructural results presented above. Within the present material system, the intermediate resin content was associated with low measured porosity, high measured flexural properties, and high impact-energy absorption, which may have contributed to more effective load redistribution during ballistic impact.

BFS provides a quantitative measure of rear-face deformation and is an important comparative indicator for armor performance. However, BFS alone should not be interpreted as a direct measure of wearer safety because the present study did not include a human surrogate, biomechanical injury assessment, or a validated behind-armor blunt-trauma criterion. Therefore, the measured BFS values are reported here primarily for a comparative evaluation of the three armor configurations.

#### 3.3.2. Ceramic Faceplate Damage Behavior

Figure 11a–c depicts the typical failure patterns on the rear faces of the ceramic tiles supported by the three backing schemes. Group B induced the most symmetrical and fully developed ceramic fragmentation cone, capturing the widest fracture footprint and uniform crack distribution. Group A led to localized, severe ceramic pulverization confined to the impact core, leaving the perimeter mostly intact. Group C generated an intermediate pattern with distinct brittle macro-cleavage cracks. Figure 11d illustrates the geometric definition of the ceramic fragmentation cone and the corresponding semi-cone angle. Table 6 presents the size of the ceramic fragmentation semi-cone angle and the number of cracks corresponding to different support layers. By analyzing the relationship between the ceramic semi-cone angle and the flexural strength and modulus of the support layer across the three resin-content configurations, the semi-cone angle exhibited an apparent positive association with the measured flexural strength and flexural modulus of the support layer. Specifically, the largest semi-cone angle was observed for Group B, which also exhibited the highest measured flexural strength and modulus. This group-level trend is consistent with the proposed role of support-layer stiffness and load redistribution in influencing the development of ceramic fracture, and the corresponding relationship is shown in Figure 12. However, given the limited number of resin-content levels and the absence of replicate-level statistical distributions for all mechanical properties, this observation should be interpreted as an experimental association rather than evidence of a causal relationship.

To further quantify the variability in ceramic damage morphology, the statistical characteristics of the semi-cone angle and radial crack count are summarized in Table 7 and Table 8, respectively. The mean semi-cone angles were 75.40 ± 0.85°, 80.81 ± 0.16°, and 79.62 ± 0.46° for Groups A, B, and C, respectively. One-way ANOVA indicated a significant difference among the three groups (F = 102.116, *p* < 0.001), and all three pairwise comparisons remained significant after Holm correction (Table 8). Thus, the ceramic fracture-cone geometry varied systematically among the three resin-content configurations under the present ballistic conditions.

The radial crack counts were 7.75 ± 0.96, 11.75 ± 1.50, and 10.50 ± 1.29 for Groups A, B, and C, respectively. The overall ANOVA was also significant (F = 10.397, *p* = 0.0046). Pairwise analysis showed significant differences between A and B (Holm-adjusted *p* = 0.0184) and between A and C (Holm-adjusted *p* = 0.0320), whereas the difference between B and C was not statistically significant (Holm-adjusted *p* = 0.2544).

By analyzing the relationship between the ceramic semi-cone angle and the flexural strength and modulus of the support layer, the present results suggest an association between the mechanical characteristics of the support layer and the resulting ceramic fracture-cone geometry. However, because only three resin-content levels were investigated and the mechanical-property measurements were not obtained from the same replicate specimens used for ballistic testing, the available data do not permit a quantitative assessment of the correlation or causal relationship between support-layer mechanical properties and ceramic fracture morphology. Therefore, this relationship is interpreted as a group-level experimental association that provides mechanistic context for the observed ballistic response, rather than as a quantitatively established predictive relationship.

When the resin content was 20 wt%, the relatively low resin content was accompanied by weaker interfacial integrity and lower measured flexural performance. As the resin content increased to 30 wt%, the composite exhibited higher measured flexural strength and modulus together with the largest measured semi-cone angle. When the resin content increased to 38 wt%, the semi-cone angle remained high but decreased relative to Group B. These observations indicate that an intermediate resin content was associated with the most developed ceramic fragmentation pattern within the investigated parameter range.

#### 3.3.3. Support Layer Failure Modes and Energy Dissipation Mechanisms

To enable a clear assessment of how far the projectile penetrates into each functional layer of the composite armor, Table 9 provides a comprehensive summary of the depth of penetration (DOP) data corresponding specifically to the support layer and the backing layer, based on the results of the ballistic tests.

Based on macroscopic and microscopic damage metrics, three distinct penetration failure modes were identified for the ceramic composite armor. Figure 13 presents the post-impact failure morphology of the Aramid III support layer containing only 20 wt% resin. As shown in Figure 13a, the strike face exhibited severe wrinkling and highly localized damage, suggesting less effective load redistribution under the investigated impact condition throughout the panel. The CT image in Figure 13b reveals incomplete resin impregnation and insufficient bonding between neighboring fiber bundles, which is consistent with the relatively poor load-transfer capability of the support layer. Owing to the low resin content, interfacial load transfer between adjacent yarn bundles was likely limited. Under projectile impact, only the fiber bundles located directly along the penetration path appeared to carry a larger proportion of the local load, while the surrounding fibers suggesting limited participation of the surrounding fiber bundles. The lack of sufficient matrix constraint may have facilitated local yarn separation and localized penetration, resulting in a drill-through-dominated failure mode. Consequently, the support layer failed to develop a large-area load-bearing response, resulting in low energy-absorption efficiency and a relatively high back-face signature. These observations indicate that the low-resin configuration was unable to effectively redistribute impact loads or activate large-scale fiber deformation, leading to inferior ballistic performance.

As shown in Figure 14a,b, the projectile was successfully arrested within the front region of the 30 wt% resin-content Aramid III support layer, and no complete perforation occurred. A broad deformation zone developed across the laminate, while the characteristic cross-shaped pattern in Figure 14a indicates that both warp and weft yarns were simultaneously engaged, enabling efficient load redistribution and the formation of a square-shaped damage zone. The CT image in Figure 14c reveals extensive matrix cracking, interlaminar delamination, and distributed fiber deformation. These observations provide morphological evidence that multiple damage mechanisms were activated simultaneously. However, the present study did not directly measure the individual energy contributions of fiber stretching, matrix cracking, delamination, or friction. As illustrated in Figure 14d, the support layer remained structurally intact and deformed synergistically with the UHMWPE energy-absorbing backing layer. The observed coupled deformation is consistent with a synergistic load-bearing response between the support and backing layers and may have contributed to the relatively low BFS measured for Group B.

The failure morphology of the 38 wt% resin-content support layer is shown in Figure 15. As illustrated in Figure 15a,b, the projectile completely perforated the support layer, producing a plugging hole accompanied by longitudinal tearing and a pronounced cross-shaped crack pattern, indicating severe localized failure. The CT image in Figure 15c shows a relatively resin-rich morphology around the fiber bundles, accompanied by localized damage. Consequently, stress concentrations may have developed at yarn crossover regions, causing premature fiber failure and crack propagation along the fiber directions. As a result, the dominant failure mode was characterized by localized shear/plugging damage rather than the broad deformation observed for Group B. Although the higher resin content provided a relatively stiff support condition, premature local failure limited the development of the large-area coupled deformation observed in Group B. As shown in Figure 15d, the residual deformation was mainly accommodated by the UHMWPE backing layer. Consequently, Group C showed an intermediate ballistic response between Groups A and B under the present test conditions.

Overall, the three failure modes—drill-through, integral synergistic deformation, and shear plugging—were identified from the observed post-impact morphology. The integral synergistic deformation mode was associated with the lowest measured BFS and complete protection in all four Group B tests. Nevertheless, the present experiments did not include instrumented projectile force histories, high-speed full-field strain measurements, or layer-resolved numerical energy partitioning. Therefore, the proposed energy-dissipation mechanisms should be regarded as morphology-based interpretations rather than quantitatively validated energy-transfer pathways.

## 4. Conclusions

This study systematically investigated the effects of resin content, weaving architecture, and manufacturing process on the mechanical properties and ballistic performance of Aramid III/epoxy composites used as the support layer in B4C ceramic composite armor systems. The major conclusions are summarized as follows:

(1) Resin content significantly influenced the microstructure and mechanical properties of the Aramid III composites. The laminate containing 30 wt% resin exhibited the lowest porosity (0.3%) and the best overall mechanical performance, with a flexural strength of 421 MPa, a flexural modulus of 34.6 GPa, and an impact energy absorption of 141.5 J, whereas the 38 wt% laminate exhibited the highest measured ILSS (28.1 MPa). These results indicate that an intermediate resin content provided a favorable balance between matrix continuity, interfacial integrity, and fiber-dominated deformation under the present test conditions.
(2) The UD composite exhibited superior mechanical and impact properties compared with the plain-woven composite at the same resin content. The impact energy absorption increased from 141.5 J for the plain-woven composite to 196 J for the UD composite, corresponding to an increase of 38.5%. These results demonstrate the important influence of fiber architecture on the coupon-level mechanical and impact response. However, because the ballistic tests in this study employed plain-woven Aramid III support layers, the present results do not provide a direct comparison of the ballistic performance of UD and plain-woven architectures.
(3) The ballistic tests showed protection rates of 50%, 100%, and 75% for Groups A, B, and C, respectively. The mean BFS values of the non-perforated specimens were 37.00 ± 5.66, 11.25 ± 2.50, and 24.33 ± 2.52 mm for Groups A, B, and C, respectively, with Group B exhibiting the lowest BFS. These results indicate that the 30 wt% resin content showed the most favorable observed ballistic response within the investigated range, particularly in terms of reducing back-face deformation.
(4) The observed failure modes included drill-through, integral synergistic deformation, and shear plugging. Group B exhibited a relatively well-developed ceramic fragmentation cone, distributed radial cracking, and coordinated deformation of the Aramid III and UHMWPE backing layers, which was associated with the lower BFS observed for Group B. Overall, the results demonstrate that resin content influenced both the ceramic damage morphology and the deformation response of the backing system under the investigated ballistic conditions.

## Figures and Tables

**Figure 1:**
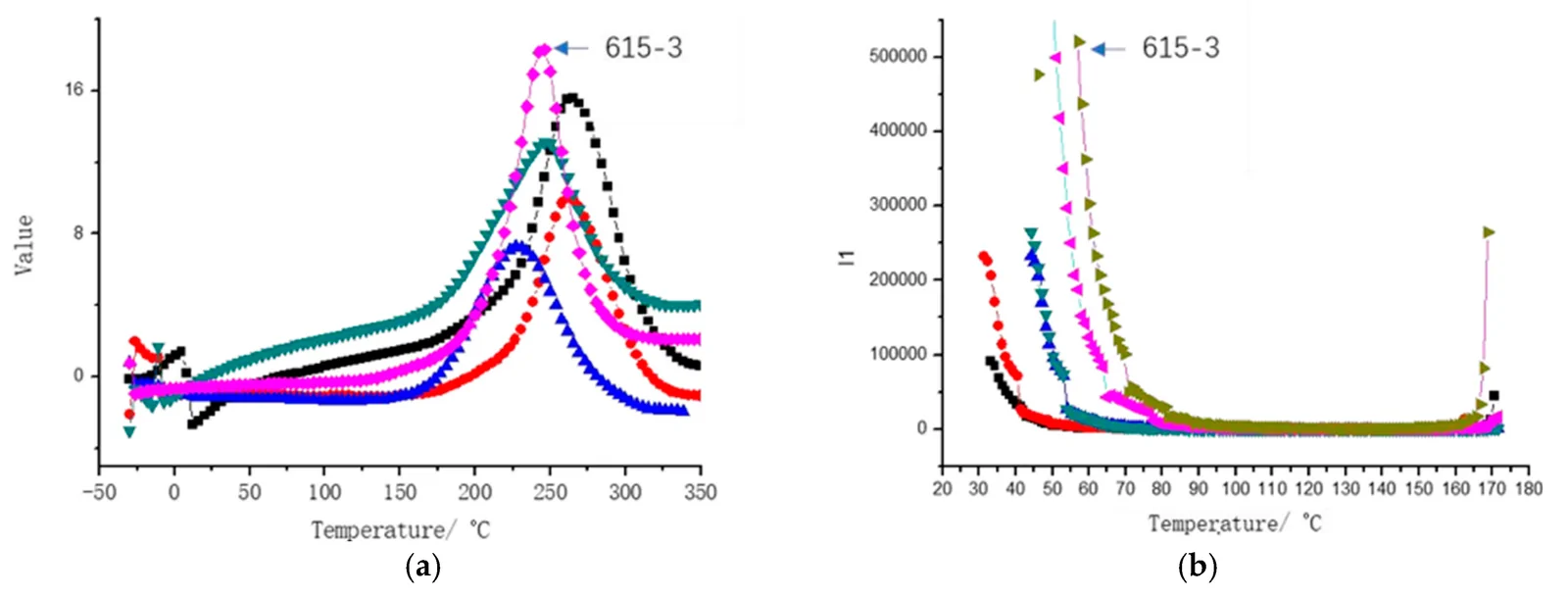
DSC curve (**a**) and viscosity–temperature curve (**b**) of toughened epoxy resin.

**Figure 2:**
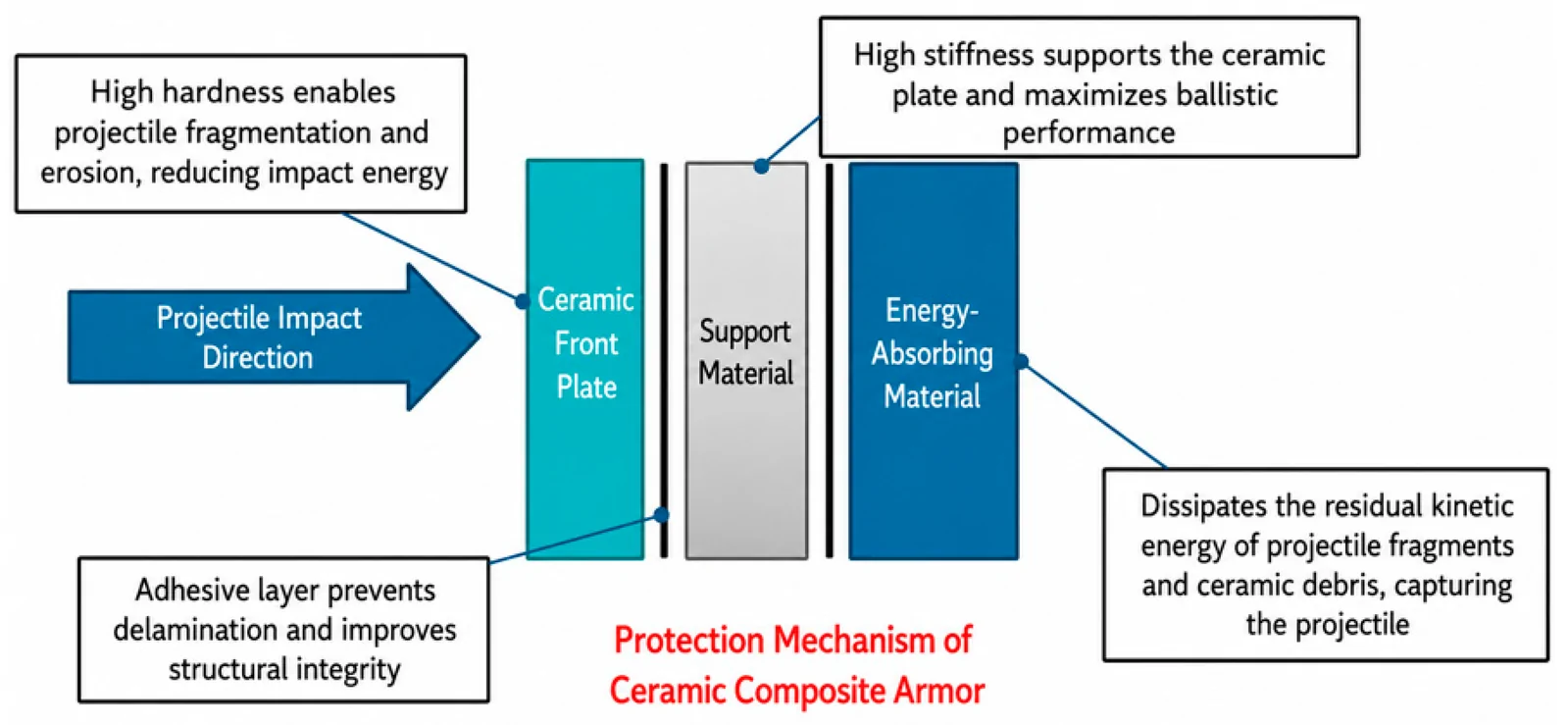
Schematic of the protection mechanism of the ceramic composite armor.

**Figure 3:**
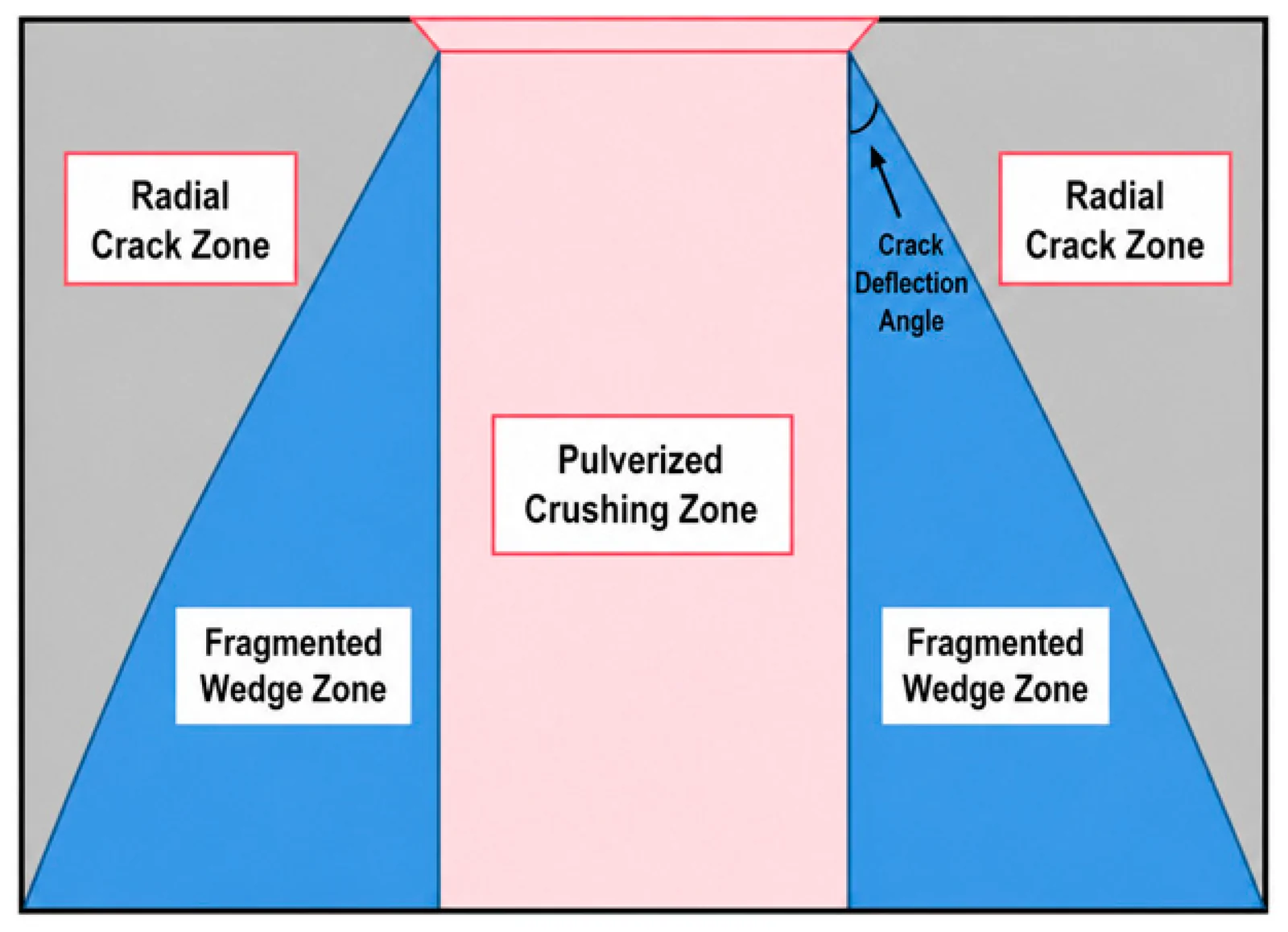
Schematic distribution of the ceramic fragmentation cone.

**Figure 4:**
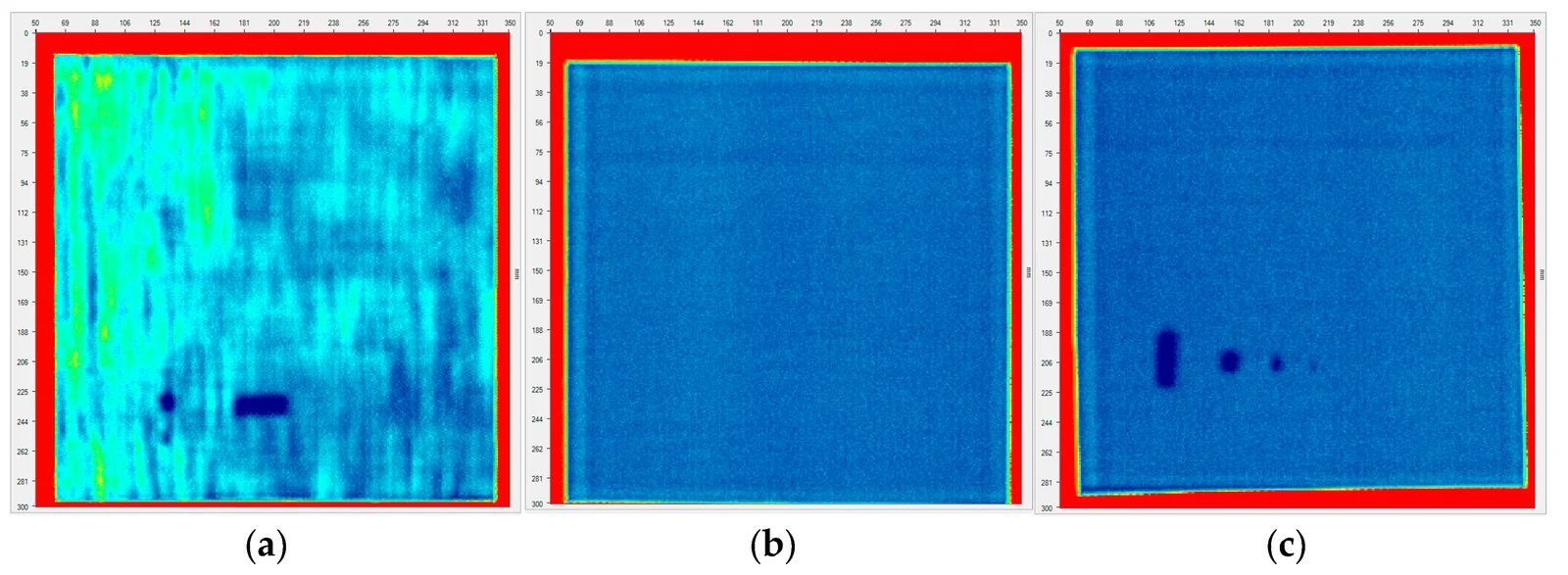
Ultrasonic C-scans of aramid composites molded by autoclave and hot press: (**a**) autoclave (20 wt%), (**b**) autoclave (30 wt%), and (**c**) hot press (30 wt%).

**Figure 5:**
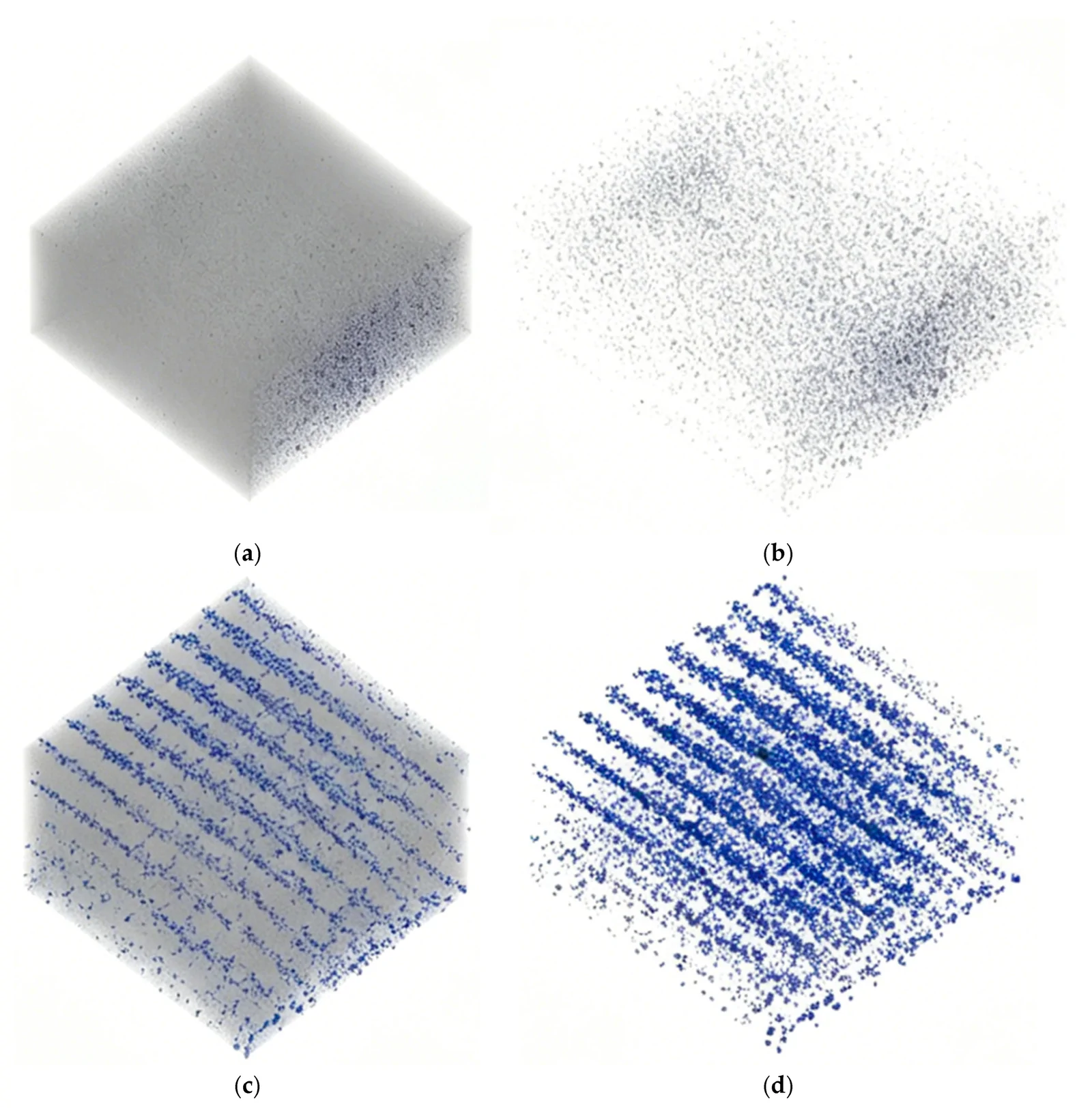
Micro-CT reconstructions of 30 wt% resin aramid composites via hot press (**a**,**b**) and autoclave (**c**,**d**) before and after rendering.

**Figure 6:**
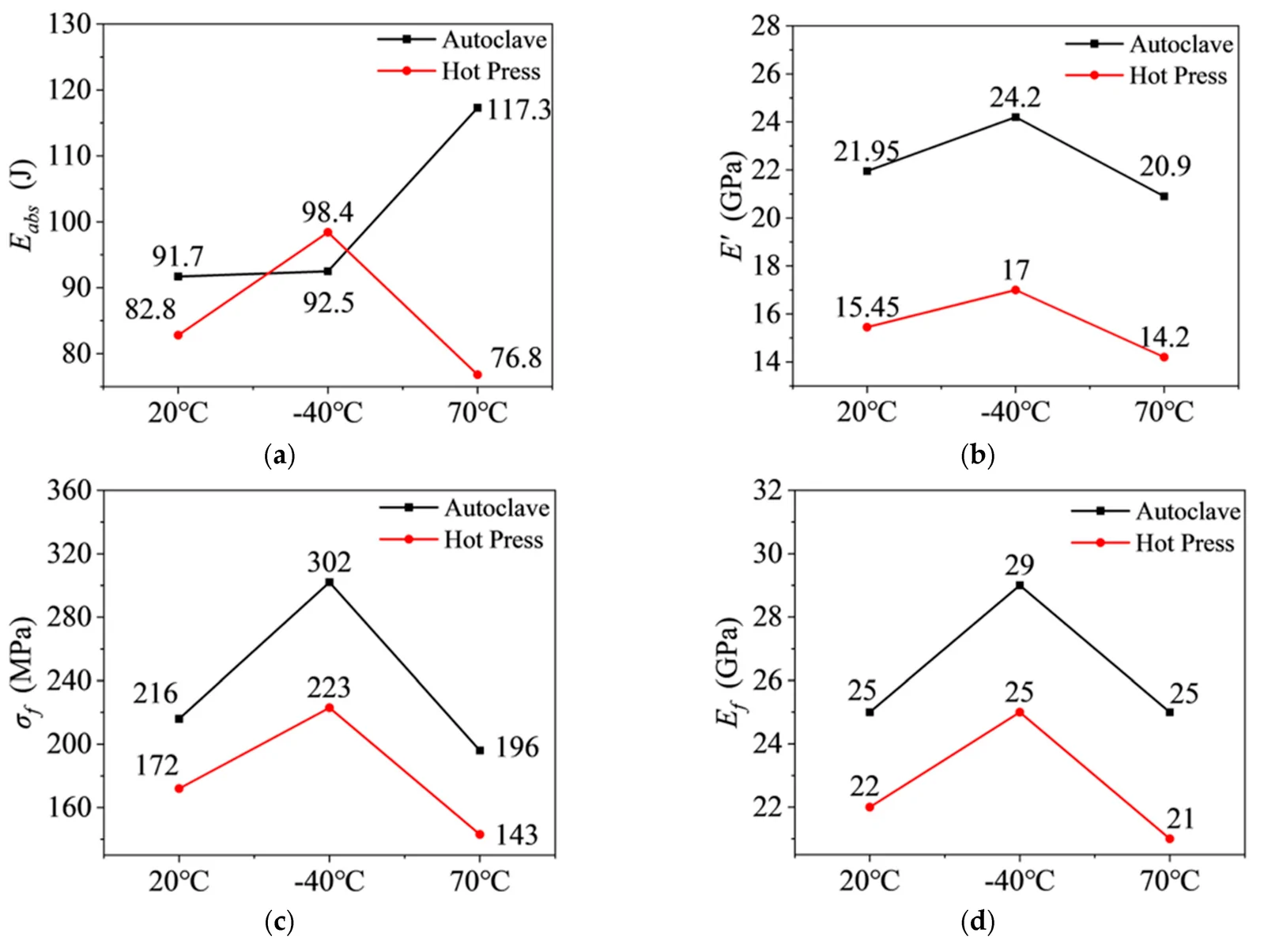
Temperature-dependent quasi-static mechanical properties: (**a**) puncture energy, (**b**) storage modulus, (**c**) flexural strength, and (**d**) flexural modulus.

**Figure 7:**
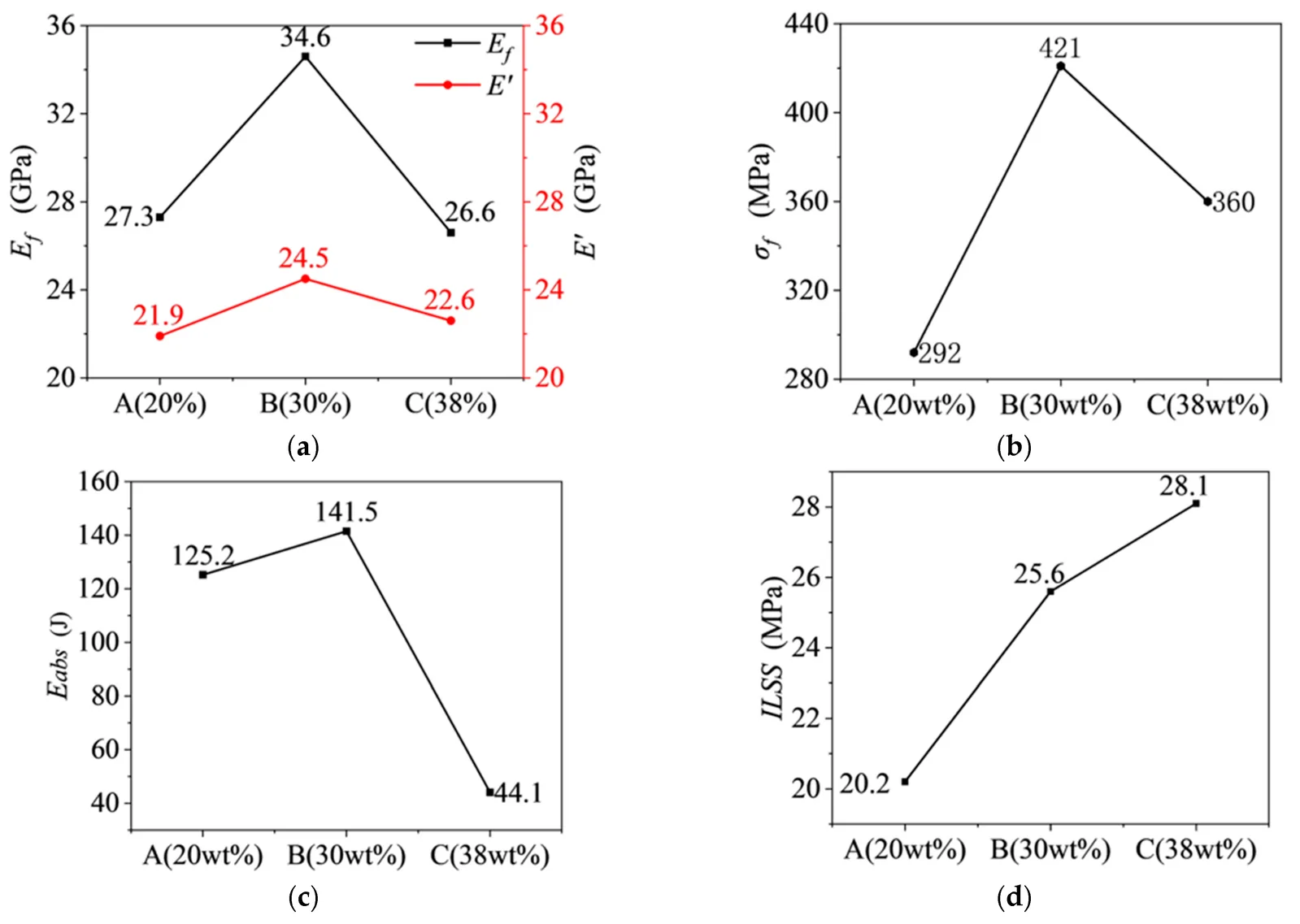
Effect of resin content on the mechanical properties of the Aramid III composites: (**a**) flexural and storage moduli, (**b**) flexural strength, (**c**) ILSS, and (**d**) drop-weight impact energy.

**Figure 8:**
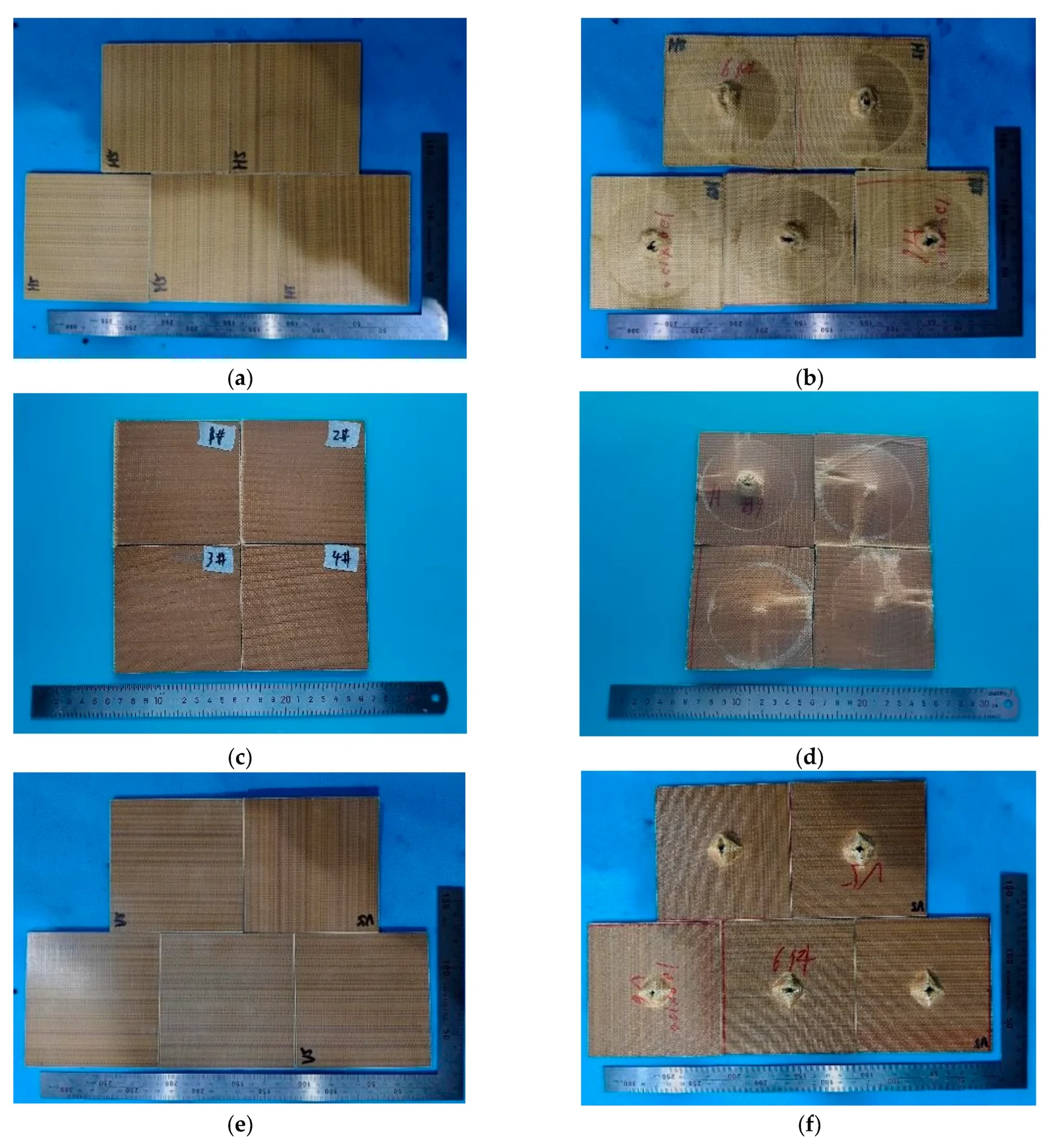
Post-impact failure morphologies of aramid composites with varying resin contents: (**a**,**b**) 20 wt%, (**c**,**d**) 30 wt%, and (**e**,**f**) 38 wt% before and after impact.

**Figure 9:**
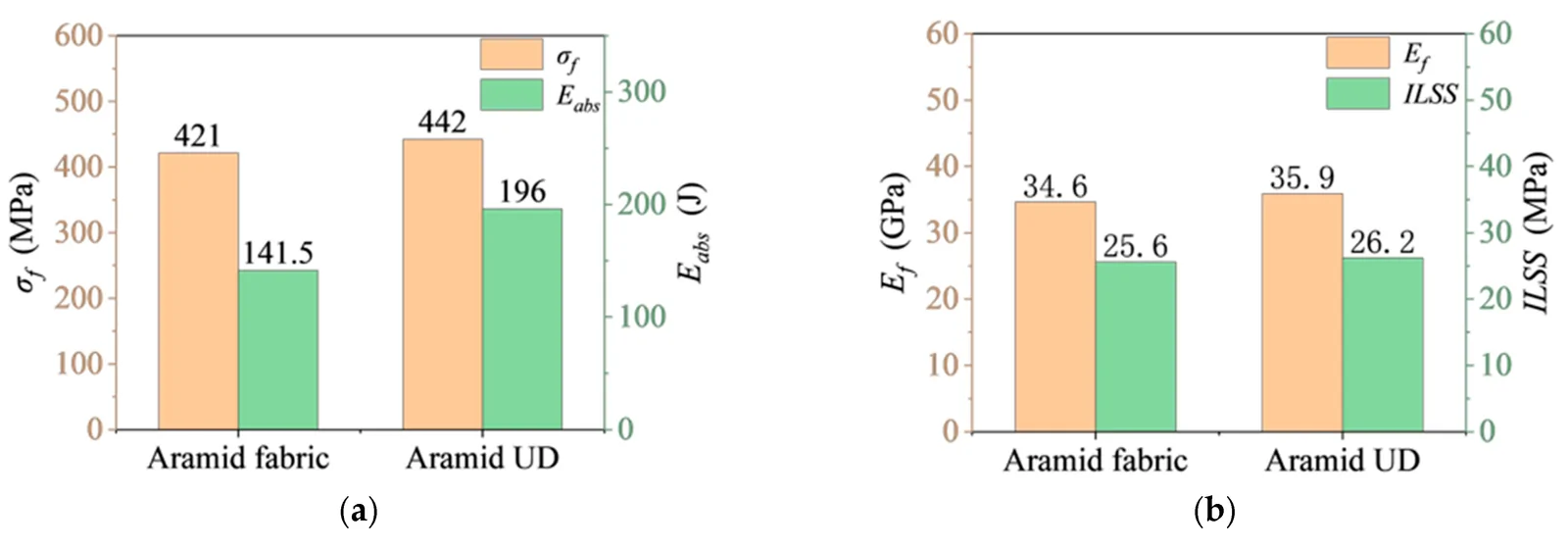
Mechanical properties of aramid UD vs. plain-woven fabric composites (30 wt% resin): (**a**) flexural strength and impact energy; (**b**) flexural modulus and ILSS.

**Figure 10:**
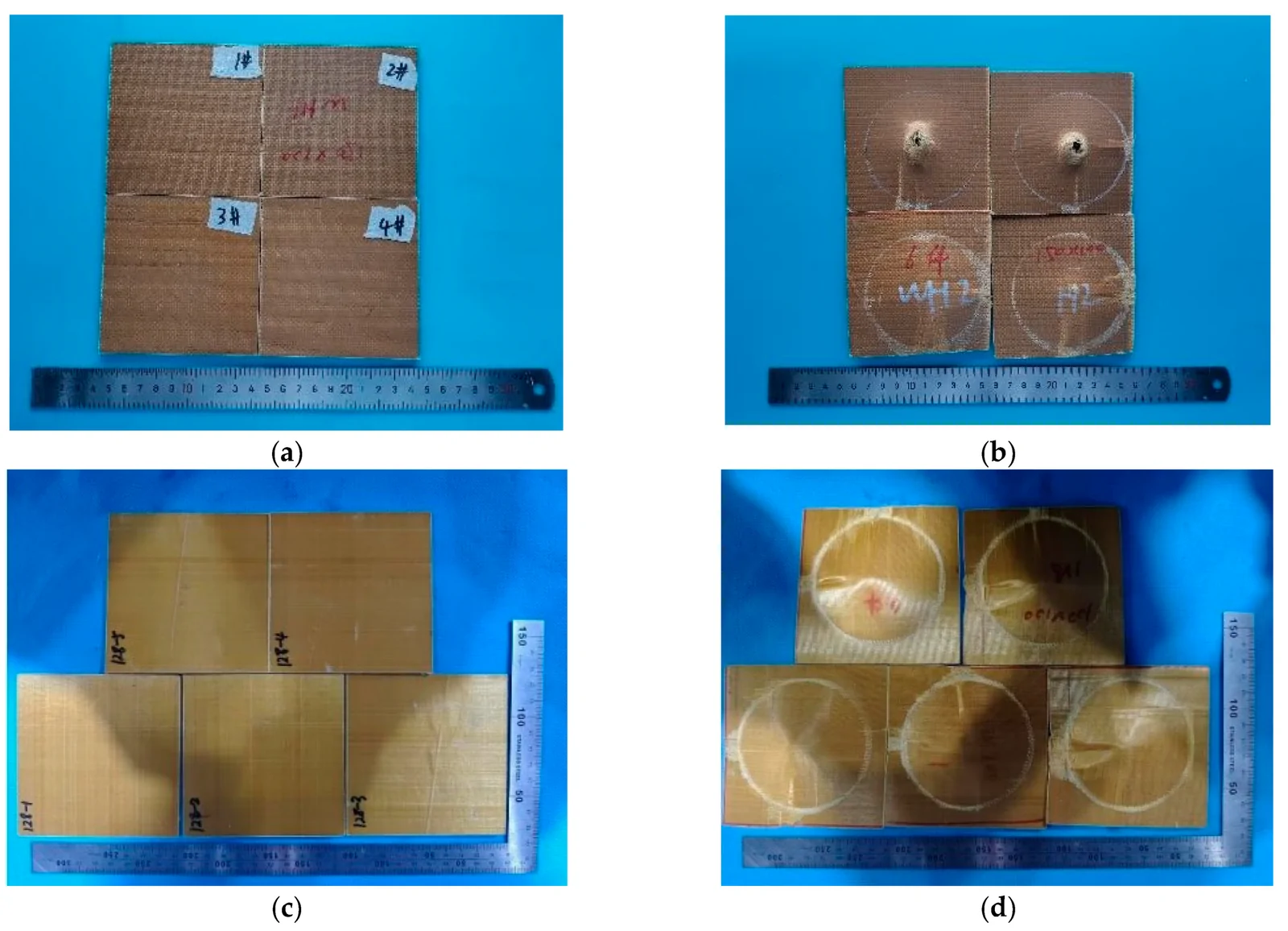
Rear-face failure morphologies of aramid composites with different weave architectures: (**a**,**b**) plain-woven and (**c**,**d**) UD before and after impact.

**Figure 11:**
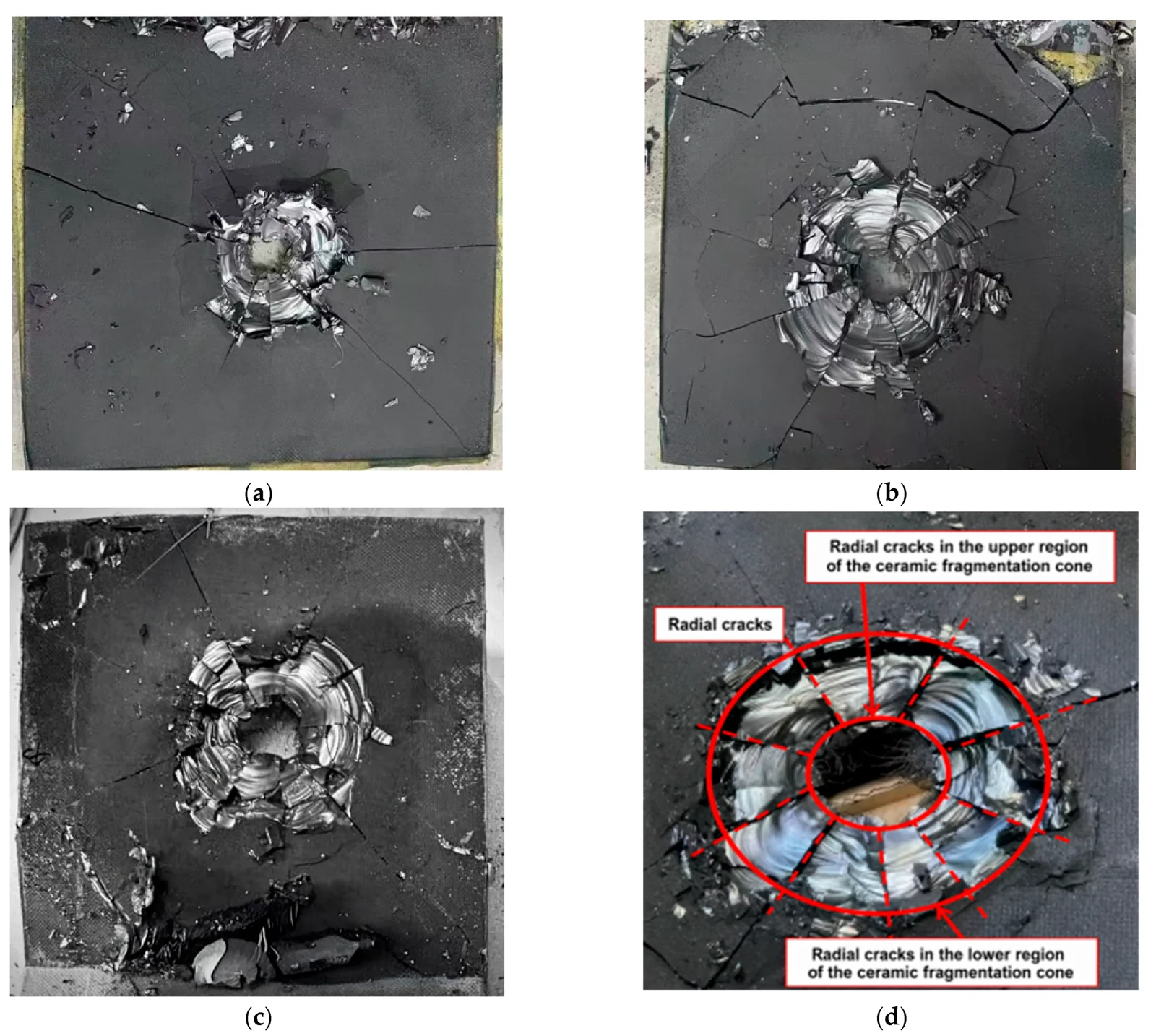
Ceramic faceplate failure morphologies and fragmentation cone partitioning: (**a**) Option A (20 wt%), (**b**) Option B (30 wt%), (**c**) Option C (38 wt%), and (**d**) zoning schematic.

**Figure 12:**
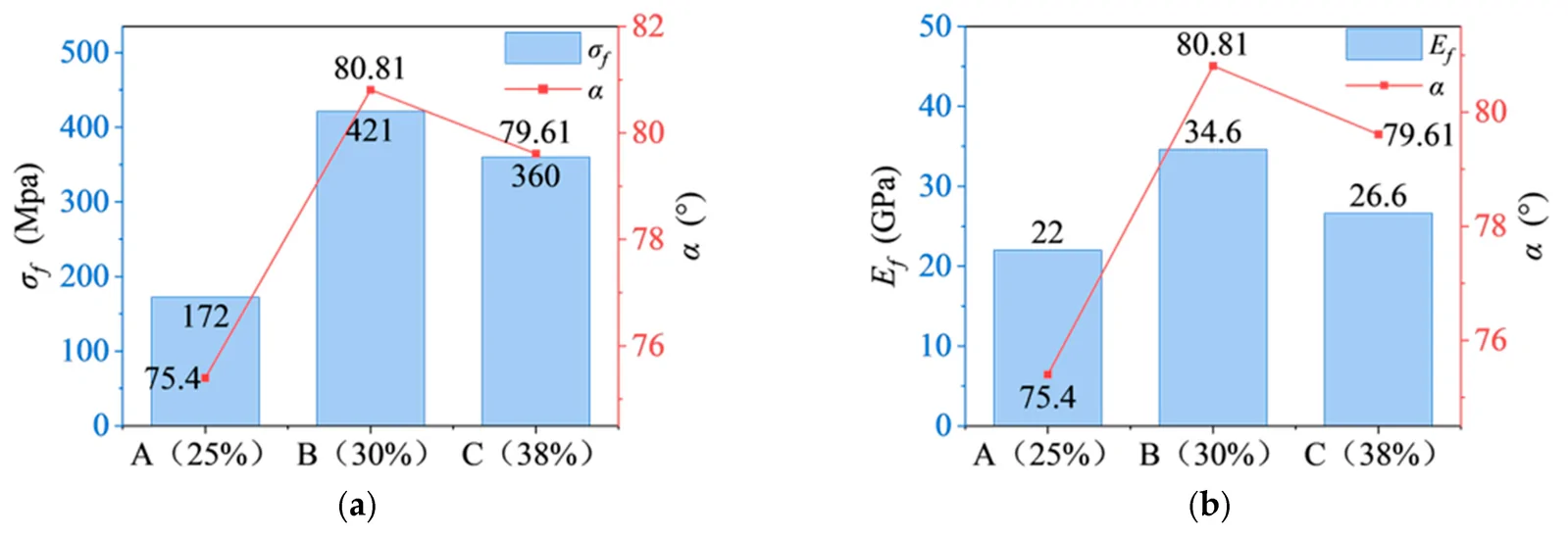
Ceramic semi-cone angle vs. support layer mechanical properties: (**a**) flexural strength and (**b**) flexural modulus.

**Figure 13:**
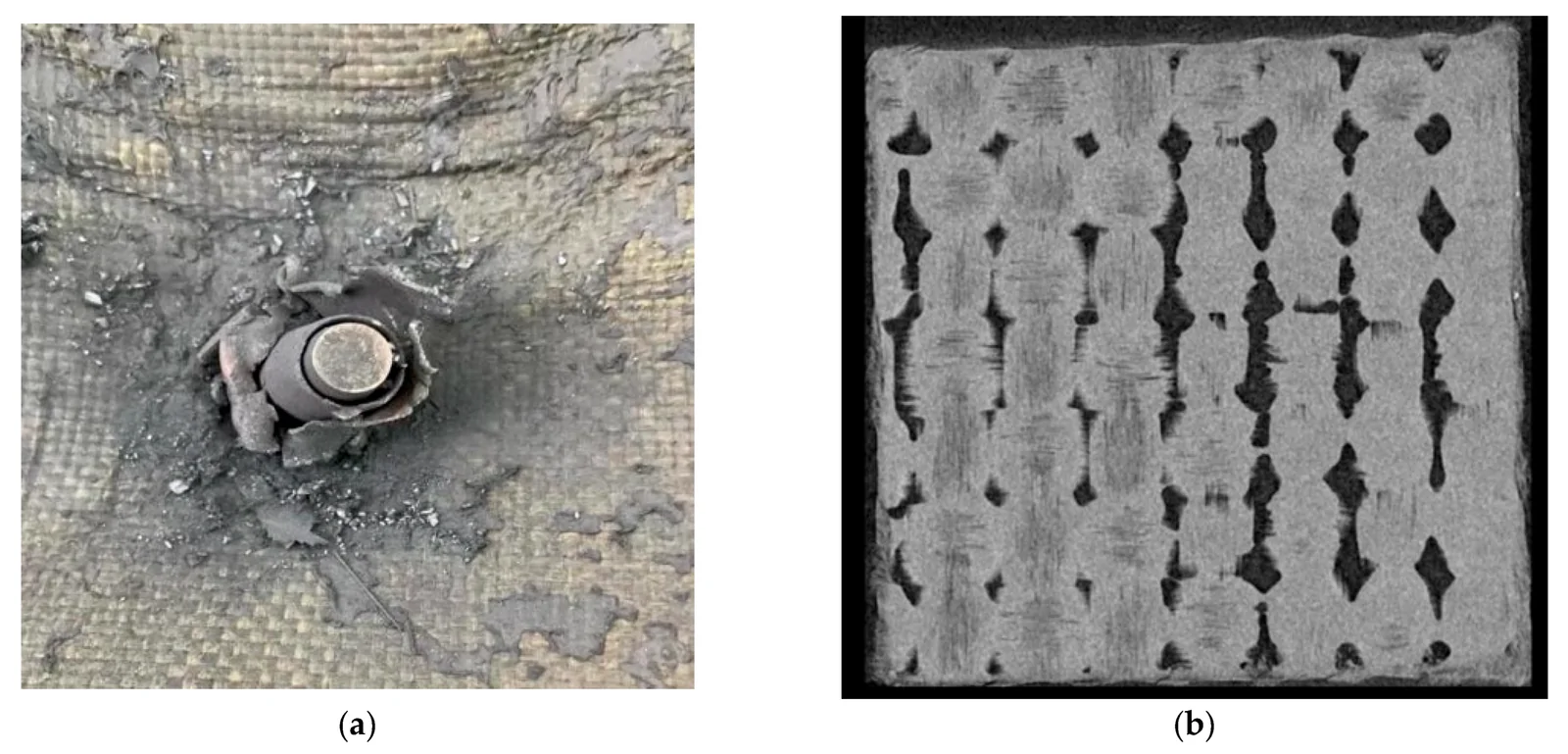
Failure morphology of Option A support layer (20 wt%): (**a**) strike face, and (**b**) CT scan.

**Figure 14:**
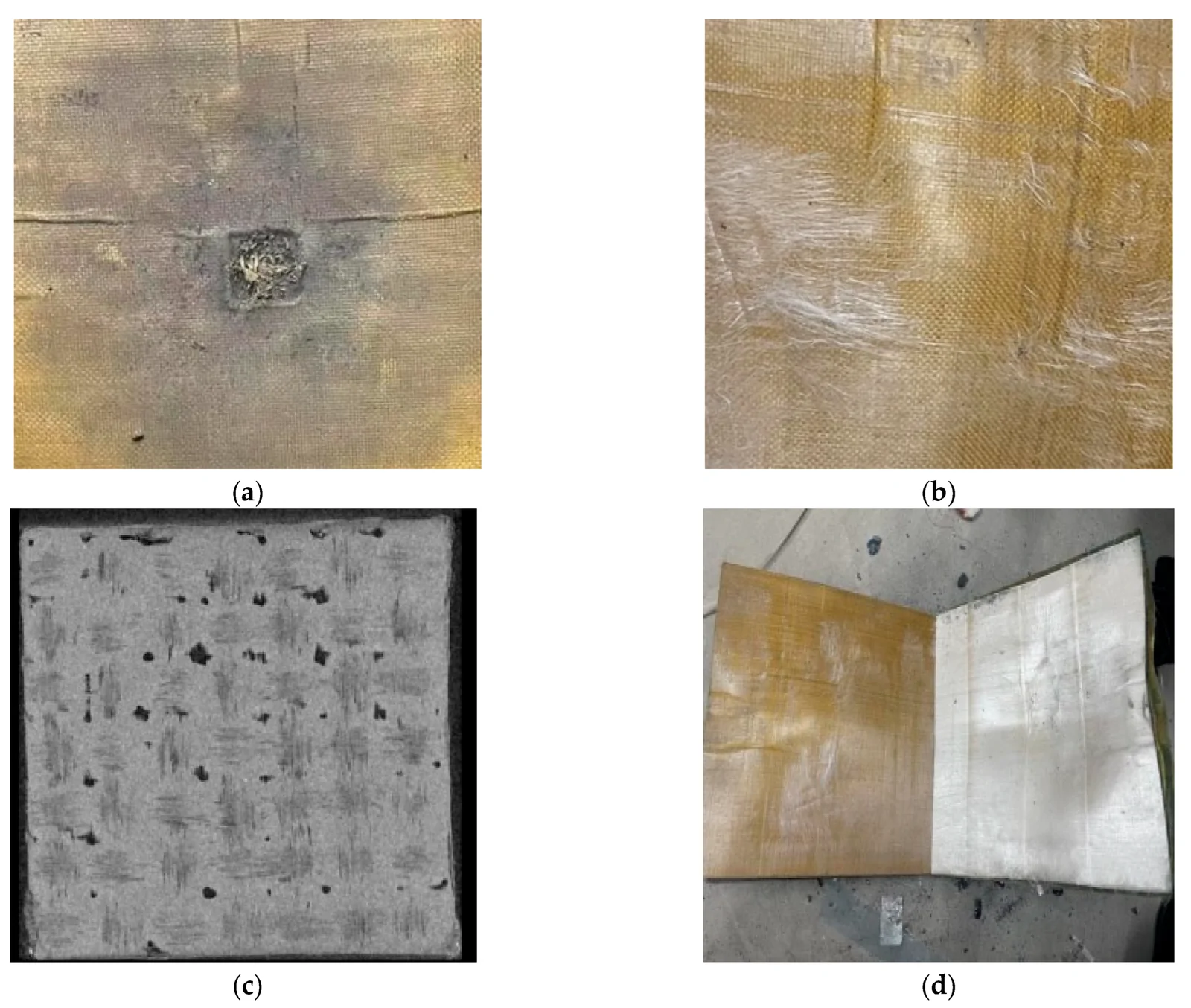
Failure morphology of Option B support layer (30 wt%): (**a**) strike face, (**b**) rear face, (**c**) CT scan, and (**d**) synergistic deformation of support and energy-absorbing layers.

**Figure 15:**
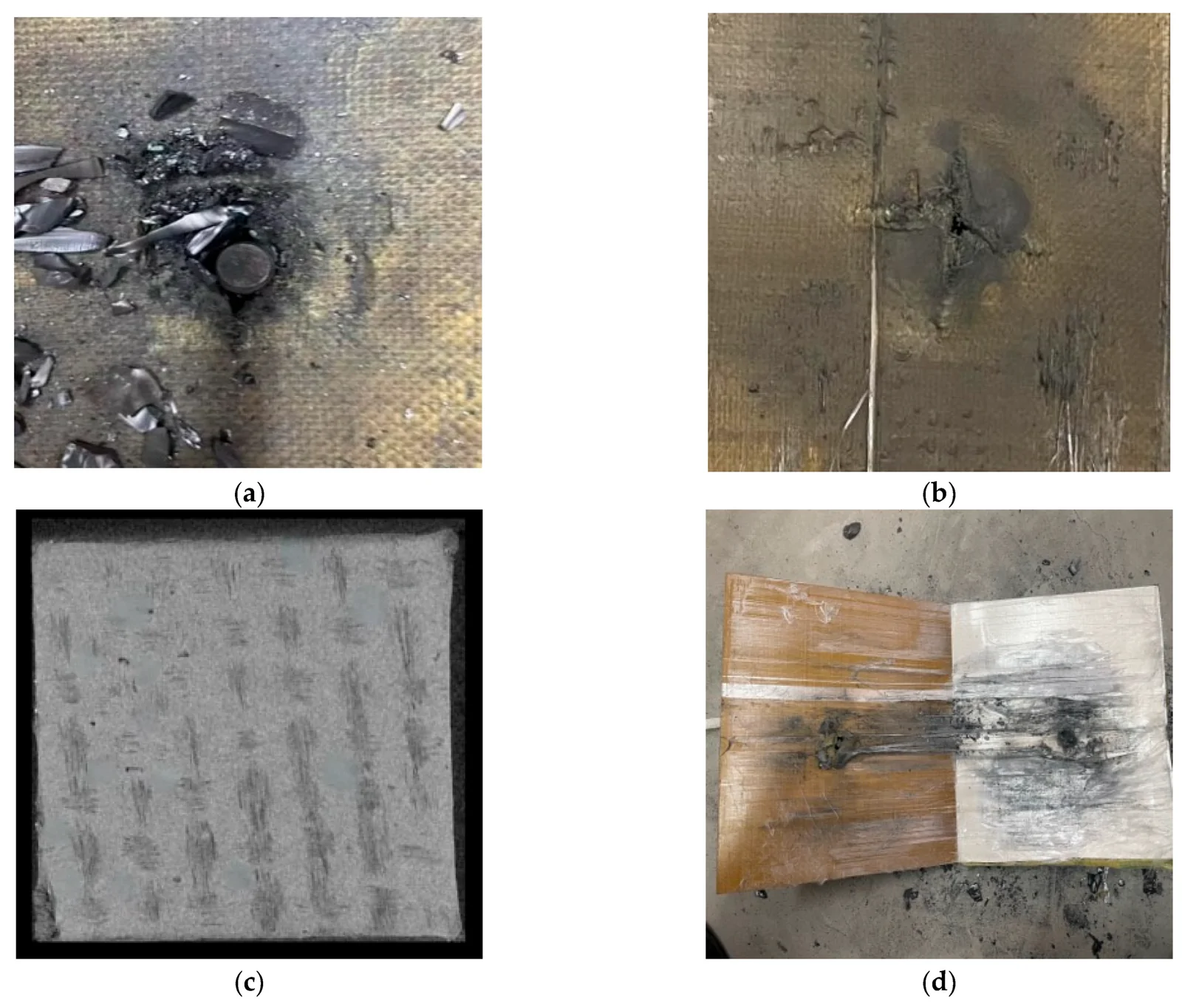
Failure morphology of Option C support layer (38 wt%): (**a**) strike face, (**b**) rear face, (**c**) CT scan, and (**d**) shear plugging of support and energy-absorbing layers.

**Table 1:** Density and porosity of Aramid III composites with different resin contents.

Resin Content (wt%)	Measured Density *ρ_exp_* (g/cm^3^)	Theoretical Density *ρ_theo_* (g/cm^3^)	Porosity *V_v_* (%)
20 (A)	1.38	1.41	1.8
30 (B)	1.40	1.40	0.3
38 (C)	1.39	1.41	1.2

**Table 2:** Summary of live-fire ballistic test results for the ceramic composite armors.

Option	Target ID	Target Weight (g)	Areal Density (kg/m^2^)	Target Thickness (mm)	Projectile Velocity (m/s)	Protection Outcome	BFS (mm)	Mean BFS (mm)
A	A1	3830	42.55	24.51	489	Protection	41	37.00
	A2	3827	42.52	24.42	497	Perforation	-	
	A3	3823	42.47	24.46	496	Perforation	-	
	A4	3826	42.51	24.52	489	Protection	33	
B	B1	3832	42.58	24.51	493	Protection	12	11.25
	B2	3828	42.53	24.44	497	Protection	14	
	B3	3836	42.62	24.52	501	Protection	11	
	B4	3830	42.56	24.43	496	Protection	8	
C	C1	3831	42.57	24.33	499	Protection	27	24.33
	C2	3826	42.51	24.42	498	Perforation	-	
	C3	3824	42.49	24.46	501	Protection	22	
	C4	3821	42.46	24.47	500	Protection	24	

**Table 3:** Statistical summary of ballistic performance.

Configuration	Resin Content (wt%)	Number of Shots	Protection, n (%)	Perforation, n (%)	Valid BFS, n	BFS (mm), Mean ± SD
A	20	4	2 (50.0)	2 (50.0)	2	37.00 ± 5.66
B	30	4	4 (100.0)	0 (0.0)	4	11.25 ± 2.50
C	38	4	3 (75.0)	1 (25.0)	3	24.33 ± 2.52

**Table 4:** Pairwise statistical comparison of BFS.

Comparison	BFS Group 1 (mm)	BFS Group 2 (mm)	Test	*p*-Value	Holm-Adjusted *p*-Value	Statistical Interpretation
A vs. B	37.00 ± 5.66	11.25 ± 2.50	Welch’s *t*-test	0.0748	0.1495	Not significant
A vs. C	37.00 ± 5.66	24.33 ± 2.52	Welch’s *t*-test	0.1618	0.1618	Not significant
B vs. C	11.25 ± 2.50	24.33 ± 2.52	Welch’s *t*-test	0.0016	0.0048	Significant

**Table 5:** Statistical comparison of ballistic protection rates.

Comparison	Protection Rate	Statistical Test	Two-Sided *p*-Value	Interpretation
A vs. B	50.0% vs. 100.0%	Fisher’s exact test	0.4286	Not significant
A vs. C	50.0% vs. 75.0%	Fisher’s exact test	1.0000	Not significant
B vs. C	100.0% vs. 75.0%	Fisher’s exact test	1.0000	Not significant

**Table 6:** Ceramic fragmentation cone parameters and radial crack statistics.

Option	Target ID	Cone Top Diameter *D* (mm)	Cone Base Diameter *d* (mm)	Semi-Cone Angle (°)	Mean Semi-Cone Angle	Protection Outcome	Radial Crack Count
A	A1	32.3	112.5	76.04	75.40	Protection	7
	A2	35.3	106.2	74.28		Perforation	8
	A3	31.2	106.7	75.20		Perforation	7
	A4	29.1	109.5	76.07		Protection	9
B	B1	36.2	158.3	80.74	80.81	Protection	10
	B2	38.5	159.2	80.63		Protection	13
	B3	37.9	162.1	80.89		Protection	11
	B4	40.1	165.6	80.99		Protection	13
C	C1	36.8	132.2	79.36	79.62	Protection	12
	C2	39.6	132.8	79.11		Protection	9
	C3	39.5	140.2	79.91		Protection	11
	C4	38.9	141.4	80.08		Protection	10

**Table 7:** Statistical summary of the ceramic fragmentation characteristics.

Configuration	Resin Content (wt%)	n	Semi-Cone Angle (°), Mean ± SD	95% CI (°)	Radial Crack Count, Mean ± SD	95% CI
A	20	4	75.40 ± 0.85	74.05–76.75	7.75 ± 0.96	6.23–9.27
B	30	4	80.81 ± 0.16	80.56–81.07	11.75 ± 1.50	9.36–14.14
C	38	4	79.62 ± 0.46	78.89–80.34	10.50 ± 1.29	8.45–12.55

**Table 8:** ANOVA and pairwise statistical analysis of the ceramic fragmentation semi-cone angle.

Analysis	Comparison	Mean Difference (°)	Test Statistic	Raw *p*	Holm-Adjusted *p*	Significance
One-way ANOVA	A vs. B vs. C	—	F = 102.116	<0.001	—	Significant
Pairwise Welch *t*-test	A vs. B	−5.4125	t ≈ −12.07	1.56 × 10^−5^	4.68 × 10^−5^	Significant
Pairwise Welch *t*-test	A vs. C	−4.2150	t ≈ −8.93	1.22 × 10^−4^	2.44 × 10^−4^	Significant
Pairwise Welch *t*-test	B vs. C	1.1975	t ≈ 4.08	2.55 × 10^−3^	2.55 × 10^−3^	Significant

**Table 9:** Penetration depth across different backing configurations.

Option	Target ID	Aramid Penetration DOP	UHMWPE Penetration DOP	BFS (mm)	Protection Outcome
A	A1	Complete Perforation	2 mm	41	Protection
	A2	Complete Perforation	Complete Perforation	-	Perforation
	A3	Complete Perforation	Complete Perforation	-	Perforation
	A4	Complete Perforation	3 mm	33	Protection
B	B1	3 mm	Deformation Only	12	Protection
	B2	4 mm	Deformation Only	14	Protection
	B3	4 mm	Deformation Only	11	Protection
	B4	2 mm	Deformation Only	8	Protection
C	C1	Complete Perforation	2 mm	27	Protection
	C2	Complete Perforation	Complete Perforation	-	Perforation
	C3	Complete Perforation	2 mm	22	Protection
	C4	Complete Perforation	3 mm	24	Protection

## Data Availability

The data presented in this study are available on request from the corresponding authors.
